# Systematic Analysis of Impact of Sampling Regions and Storage Methods on Fecal Gut Microbiome and Metabolome Profiles

**DOI:** 10.1128/mSphere.00763-19

**Published:** 2020-01-08

**Authors:** Yali Liang, Tianyu Dong, Minjian Chen, Lianping He, Tingzhang Wang, Xingyin Liu, Hang Chang, Jian-Hua Mao, Bo Hang, Antoine M. Snijders, Yankai Xia

**Affiliations:** a School of Public Health, Wannan Medical College, Wuhu, China; b State Key Laboratory of Reproductive Medicine, Center for Global Health, School of Public Health, Nanjing Medical University, Nanjing, China; c Key Laboratory of Modern Toxicology of Ministry of Education, School of Public Health, Nanjing Medical University, Nanjing, China; d Department of Immunology, Nanjing Medical University, Nanjing, China; e Zhejiang Institute of Microbiology, Key Laboratory of Microbial Technology and Bioinformatics of Zhejiang Province, Hangzhou, China; f Department of Pathogen Biology-Microbiology Division, Nanjing Medical University, Nanjing, China; g Biological Systems and Engineering Division, Lawrence Berkeley National Laboratory, Berkeley, California, USA; University of Michigan—Ann Arbor

**Keywords:** feces, metabolome, microbiome, sampling regions, storage methods

## Abstract

The contribution of human gastrointestinal (GI) microbiota and metabolites to host health has recently become much clearer. However, many confounding factors can influence the accuracy of gut microbiome and metabolome studies, resulting in inconsistencies in published results. In this study, we systematically investigated the effects of fecal sampling regions and storage and retrieval conditions on gut microbiome and metabolite profiles from three healthy children. Our analysis indicated that compared to homogenized and snap-frozen samples (standard control [SC]), different sampling regions did not affect microbial community alpha diversity, while a total of 22 of 176 identified metabolites varied significantly across different sampling regions. In contrast, storage conditions significantly influenced the microbiome and metabolome. Short-term room temperature storage had a minimal effect on the microbiome and metabolome profiles. Sample storage in RNALater showed a significant level of variation in both microbiome and metabolome profiles, independent of the storage or retrieval conditions. The effect of RNALater on the metabolome was stronger than the effect on the microbiome, and individual variability between study participants outweighed the effect of RNALater on the microbiome. We conclude that homogenizing stool samples was critical for metabolomic analysis but not necessary for microbiome analysis. Short-term room temperature storage had a minimal effect on the microbiome and metabolome profiles and is recommended for short-term fecal sample storage. In addition, our study indicates that the use of RNALater as a storage medium of stool samples for microbial and metabolomic analyses is not recommended.

**IMPORTANCE** The gastrointestinal microbiome and metabolome can provide a new angle to understand the development of health and disease. Stool samples are most frequently used for large-scale cohort studies. Standardized procedures for stool sample handling and storage can be a determining factor for performing microbiome or metabolome studies. In this study, we focused on the effects of stool sampling regions and stool sample storage conditions on variations in the gut microbiome composition and metabolome profile.

## Abstract (precis)

The gastrointestinal microbiome and metabolome can provide a new angle to understand the development of health and disease. Stool samples are most frequently used for large-scale cohort studies. Standardized procedures for stool sample handling and storage can be a determining factor for performing microbiome or metabolome studies. In this study, we focused on the effects of stool sampling regions and stool sample storage conditions on variations in the gut microbiome composition and metabolome profile.

## INTRODUCTION

Over the last decade, with the rapid development of high-throughput sequencing technologies and bioinformatic tools, gastrointestinal tract microbiome studies have made great strides. The human gut microbiome is a complex and immense ecosystem of bacterial species. Human gut microbiome research has led to renewed awareness of the relationship between the microbiome and host disease, including for example colorectal cancer (1), metabolic syndrome (2), asthma (3), and central nervous system disorders (4, 5). The most frequent approach to study the gut microbiome composition is to sequence bacterial DNA extracted from stool samples (6–8). However, factors that influence microbial DNA stability can produce significant variation in the gut microbiome composition, affecting conclusions of research findings. Therefore, the investigation of different methods for stool sample handling and storage is important for microbiome studies. Moreover, decreasing oxygen concentrations from the mucosa to the lumen of the gut can result in an uneven distribution of microbes in stools (9), leading to increased variation depending on the fecal sample location that was used to obtain microbial populations. An additional problem using fecal samples as starting material is that these samples cannot be obtained “on demand” like other types of samples. For a large cohort, fecal samples may be collected in the privacy of study participants’ homes and then stored in a domestic freezer before being transported to the laboratory for analysis (10). Even if these samples were packaged in ice packs or other protective measures, thawing of the sample may become commonplace on long trips. Gorzelak et al. found no significant change in bacterial taxa when stool samples were thawed for 7 min and then snap-frozen in liquid nitrogen for no more than four cycles (11). However, other published studies showed that four or more freeze-thaw cycles (thawed for 30 min per cycle) can result in a significant distortion of microbiota profiles from sputum samples from cystic fibrosis patients (12). Carroll et al. demonstrated that the microbiota in fecal samples during a 6-month storage period at –80°C shared more identity with its host of origin than any other sample (13). Vogtmann et al. found that the bacterial community composition was stable for 96 h at room temperature in RNALater (14). However, it remains unclear whether RNALater can preserve the microbiota during freeze-thaw cycles. A better understanding of the effects of different sampling methodologies and storage conditions on the microbiome composition is required to reduce variability in microbiome analysis across large sample cohorts.

Gas chromatography-mass spectrometry (GC-MS) and liquid chromatography-mass spectrometry (LC-MS) are widely implemented for the detection of metabolites in stool samples for disease research (15, 16). Metabolome profiling is frequently conducted in conjunction with gut microbiome studies to study the microbiome’s metabolic potential (17, 18). Therefore, it is worth investigating whether sampling methods or preservation conditions are suitable for combined microbiological and metabolite studies. Previous studies, however, paid little attention to the association between sampling or stool specimen storage methods and metabolite profiles. In this study, we systematically investigated the impact of stool sampling regions and stool sample storage conditions on variations in the gut microbiota composition and metabolic profiles in stool samples from three healthy children.

## RESULTS

### Sample collection and 16S sequencing.

**FIG 1:**
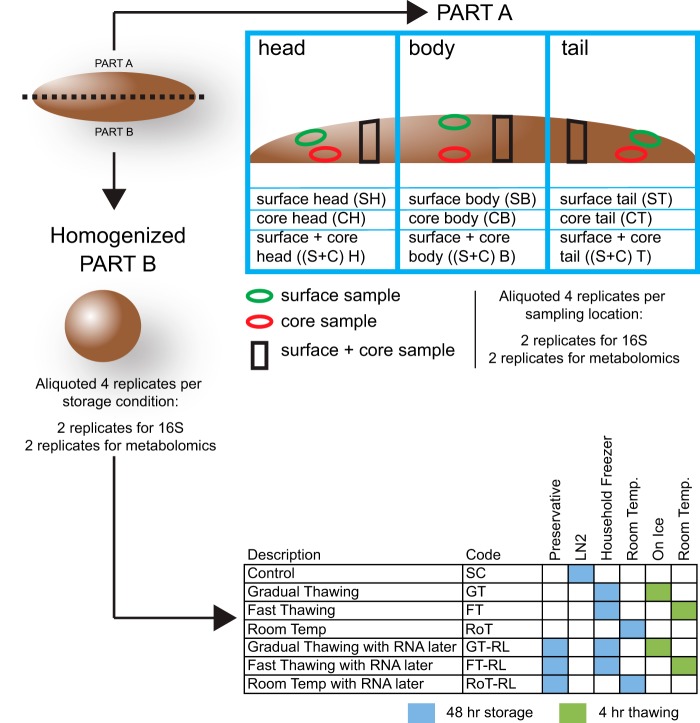
Experimental workflow. Each stool sample was divided in equal parts (parts A and B) along its longitudinal axis. Part A was used to study the effects of sampling regions on the microbiome and metabolome profiles. Part B was used to study the effects of storage and retrieval methods on the microbiome and metabolome profiles. Fecal “head” was defined as the beginning part of the discharged excrement; fecal “tail” was defined as the final part of discharged excrement; “body” was defined as the middle part of stool. “Surface and core of stool” subsamples were collected from the outside to the inside for each region.

Stool samples were collected from three healthy 34-month-old study participants from a single community nursery. A summary of the stool subsample collection is shown in Fig. 1. After dividing each stool sample into equal parts (parts A and B) along its longitudinal axis, part A was used to identify an optimal sampling location for microbiome and metabolomic analyses in the absence of homogenization. Each fecal sample was first subdivided into three parts: head, body, and tail. From each part, we then collected a surface sample, a core sample, and a combined surface and core sample. Part B was homogenized to evaluate the effects of different storage and thawing conditions on the gut microbiome community and metabolome profiles and to explore the protective effect of RNALater as a potential collecting reagent. A standard control (SC) sample, which was frozen in liquid nitrogen, was included for each fecal sample for comparison. High-throughput sequence analysis of the bacterial hypervariable V3 region of the 16S rRNA gene was conducted, and 6,536,310 raw reads from 96 stool samples were obtained. After quality-based trimming and filtering processes, 5,780,164 qualified sequences remained. All samples were rarefied to 28,265 reads, which were subsequently clustered into a total of 255 operational taxonomic units (OTUs) (see Table S1 in the supplemental material).

10.1128/mSphere.00763-19.2 TABLE S1 Summary of sequencing data and OTU table for different sampling regions and storage and retrieval methods. Download Table S1, XLSX file, 0.1 MB. Copyright © 2020 Liang et al. 2020 Liang et al. This content is distributed under the terms of the Creative Commons Attribution 4.0 International license.

### Effects of sampling regions on microbial community.

**FIG 2:**
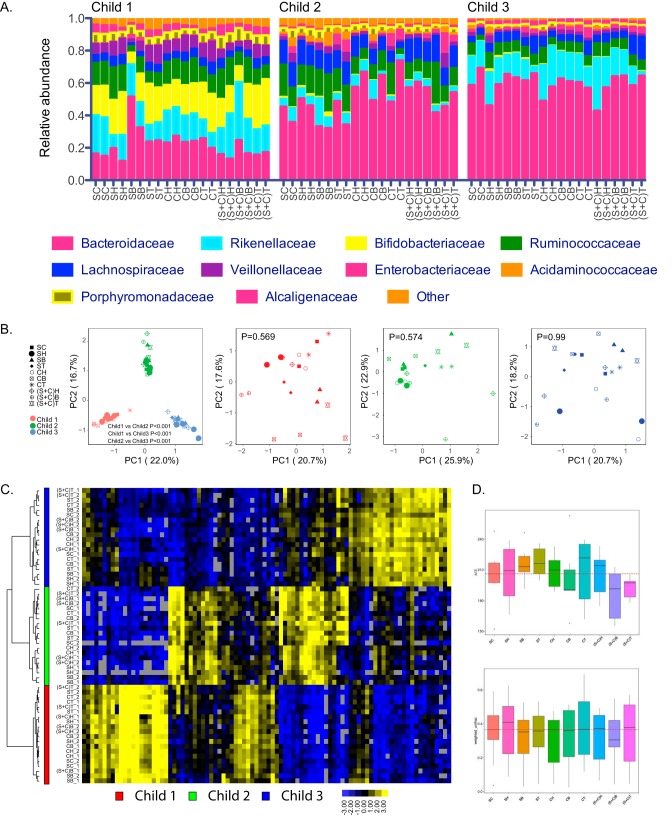
Effects of sampling regions on microbial community. (A) Relative abundance of top 10 family level taxa in samples from different sampling regions for three children. (B) Principal-component analysis of 255 OTUs across different sampling regions for individual study participants combined and individually. *P* values were obtained using PERMANOVA comparing three study participants (combined study participant PCA) or surface (SH, SB, and ST) versus core (CH, CB, and CT) samples for individual participant PCA. (C) Hierarchical clustering of 90 OTUs present in at least 80% of samples across fecal sampling sites. (D) Alpha diversity index (abundance-based coverage estimator [ACE]) (top) and beta diversity index (weighted UniFrac distance) (bottom) across different sampling regions. The red dashed lines in the graphs represent the average level of ACE index in the standard control group.

More than 96.33% of sequence reads of stool samples collected from different sampling locations on the stool samples from three children mapped to 10 family level taxa (Fig. 2A and Table S1). Principal-component analysis (PCA) of all 255 OTUs revealed that individual variability between study participants was the major driver of microbial diversity (Fig. 2B, left panel; permutational multivariate analysis of variance [PERMANOVA] *P* < 0.001), which was further confirmed by clustering analysis of 90 OTUs present in at least 80% of the samples (Fig. 2C). PCA of all 255 OTUs for each study participant individually did not show significant separation based on surface versus core samples (Fig. 2B). Next, we used three indices to estimate gut microbiota alpha diversity across sampling sites, including alpha diversity index (abundance-based coverage estimator [ACE]), Shannon, and Chao1. No significant difference in indices was observed across different sampling locations compared to SC samples (ACE, *P* = 0.104; Shannon, *P* = 0.025, adjusted *P* value [*P*_adj_] = 1.000; Chao1, *P* = 0.459; Fig. 2D, top panel; see also Fig. S1A and B in the supplemental material). For beta diversity measures, there was no significant difference in weighted UniFrac beta diversity index (Fig. 2D, bottom panel; *P* > 0.05 by Tukey honestly significant difference test [HSD]). We found no significant difference in relative abundance across different sampling locations compared to SC samples (family level q test, 0.074 ≤ *P*_adj_ ≤ 1.000; phylum level q test, 0.342 ≤ *P*_adj_ ≤ 1.000) at the family and phylum levels. Among the 50 most abundant OTUs accounting for >91.96% of total reads within sampling groups, the abundance levels of 7 OTUs were significantly different among sampling locations based on multiple comparisons (q test, *P*_adj_ < 0.05; Table S2). However, no OTUs were significantly different in any of the sampling locations in comparison to SC samples. We conclude that sampling stool samples at different regions does not have a major impact on the microbial community and their abundance levels.

10.1128/mSphere.00763-19.1 FIG S1 Microbial diversity analysis across different positions and storage and retrieval methods. (A) Box plots of Shannon index. (B) Box plots of Chao1 index. The red dotted line in the picture represents the average level of a specific index in the standard control group. The group name above a specific box plot indicated a group with statistical significance. (C) Box plots of Shannon index. (D) Box plots of Chao1 index. (E) Box plots of ACE index. The red dotted line in the picture represents the average level of a specific index in the standard control group. The group name above a specific box plot indicated a group with statistical significance. (F) Unweighted UniFrac distance. (G) Weighted UniFrac distance. The red dotted line in the picture represents the average level of a specific index in the standard control group. The group name above a specific box plot indicated a group with statistical significance. (H) UPGMA (unweighted pair group method with arithmetic mean) clustering tree for storage methods study based on weighted UniFrac distance. Asterisks indicated statistical significance as follows: *, *P*_adj_ < 0.05; **, *P*_adj_ < 0.01; ***, *P*_adj_ < 0.001. Download FIG S1, PDF file, 2.0 MB. Copyright © 2020 Liang et al. 2020 Liang et al. This content is distributed under the terms of the Creative Commons Attribution 4.0 International license.

10.1128/mSphere.00763-19.3 TABLE S2 OTUs with differential abundance across different sampling regions. Download Table S2, XLSX file, 0.01 MB. Copyright © 2020 Liang et al. 2020 Liang et al. This content is distributed under the terms of the Creative Commons Attribution 4.0 International license.

### Effects of storage and retrieval methods on microbial community.

**FIG 3:**
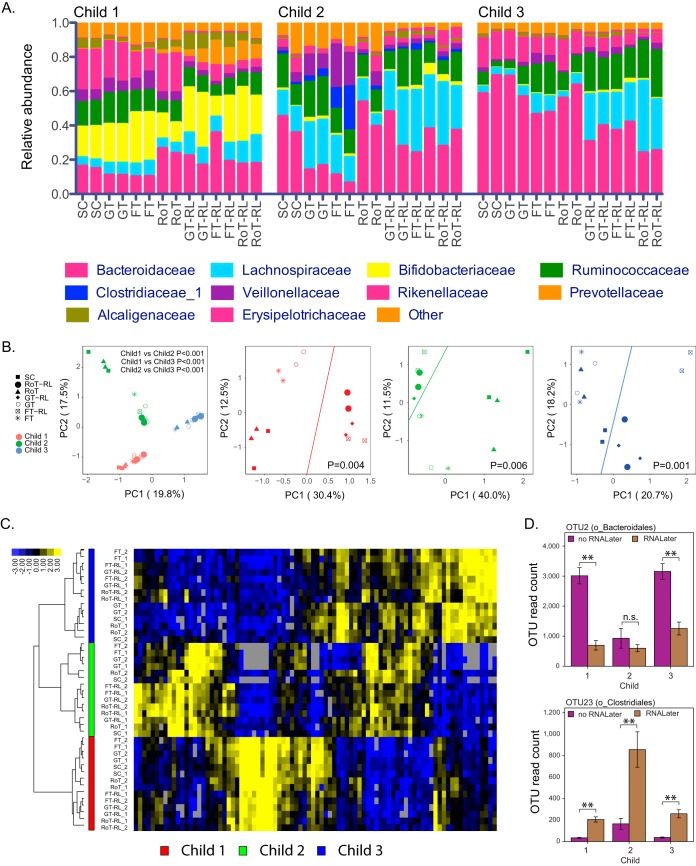
Effects of storage and retrieval methods on microbial community. (A) The relative abundance of top 10 family level taxa using different storage and retrieval methods for three children. (B) Principal-component analysis of 255 OTUs across different storage and retrieval methods for individual study participants combined and individually. *P* values were obtained using PERMANOVA comparing three study participants (combined study participant PCA) or RNALater storage (RoT-RL, GT-RL, and FT-RL) versus no RNALater storage (RoT, GT, FT, SC) samples for individual participant PCA. (C) Hierarchical clustering of 86 OTUs present in at least 80% of samples across storage and retrieval methods. (D) OTU read count for OTU2 (top) and OTU23 (bottom) for individual children’s samples separated by RNALater storage condition. Error bars represent standard errors. Statistical significance is indicated as follows: *, *P*_adj_ < 0.05; **, *P*_adj_ < 0.01; n.s., not significant.

The relative abundance of the 10 most abundant taxa across different storage and retrieval methods is shown in Fig. 3A. Similar to the effect of sampling regions, PCA of all 255 OTUs across different storage and retrieval methods revealed that individual variability between study participants was the major driver of microbial community composition (Fig. 3B; PERMANOVA *P* < 0.001), which was further confirmed by clustering analysis of 86 OTUs present in at least 80% (Fig. 3C). The impacts of storage methods on alpha diversity were not significantly different compared to SC samples (ACE, *P* = 0.456; Shannon, *P* = 0.257; Chao1, *P* = 0.301; Fig. S1C to E). Of the beta diversity measures, there was no significant difference in weighted and unweighted UniFrac distances between different storage and retrieval methods compared to SC samples (weighted UniFrac, 0.174 ≤ *P*_adj_ ≤ 0.987; unweighted UniFrac, 0.254 ≤ *P*_adj_ ≤ 0.991; Tukey HSD; Fig. S1F and G). Hierarchical clustering analysis based on weighted UniFrac distance showed that room temperature (RoT) samples were closer to SC samples compared to other storage and retrieval methods and that subsamples stored in RNALater clustered together (Fig. S1H).

We observed no difference in relative abundance of samples stored at RoT or samples that were retrieved with gradual thawing (GT) and fast thawing (FT) compared with SC samples at the phylum and family levels (family and phylum level q test, 0.105 ≤ *P*_adj_ ≤ 1.000). We further analyzed the differences in relative abundance of the top 50 OTUs (accounting for 88.17% of total reads within the storage method groups) across RoT, GT, and FT samples. Among the 50 most abundant OTUs, the abundance levels of 20 OTUs were significantly different among different storage and retrieval methods based on multiple comparisons (q test, *P*_adj_ <0.05; Table S3). We observed no difference in the relative abundance of OTUs of RoT samples or samples that were retrieved with GT compared with SC samples (RoT versus SC, 0.945 ≤ *P*_adj_ ≤ 1.000; GT versus SC, 0.485 ≤ *P*_adj_ ≤ 1.000, q test). On the other hand, six OTUs were significantly different in at least one sample stored in RNALater compared to SC samples, suggesting that RNALater had a more profound effect on the microbiome composition (Table S3). To study the effect of RNALater on OTU abundance levels in more detail, we grouped samples that were stored in RNALater and compared the abundance of the 50 most abundant OTUs to the remaining samples not stored in RNALater for each child separately. The abundance levels of 18 OTUs were significantly lower in samples stored in RNALater, whereas 16 OTUs were significantly more abundant compared to samples not stored in RNALater (Fig. 3D and Table S4). Of the 18 OTUs that were decreased after storage in RNALater, 11 belonged to the order *Bacteroidales*, whereas 11 of the 16 OTUs whose abundance levels were increased after storage in RNALater belonged to the order *Clostridiales.* OTU8 and OTU23 belonged to the *Lachnospiraceae* family (order *Clostridiales*) and were significantly increased after storage in RNALater in all three children (Fig. 3D and Table S4). In conclusion, these results indicate that fecal sample storage for 48 h at room temperature or in a household freezer followed by gradual thawing does not significantly alter the microbial composition. However, storage in RNALater significantly alters the abundance levels of specific bacteria especially those belonging to the *Bacteroidales* and *Clostridiales* orders, although individual variability outweighed this effect.

10.1128/mSphere.00763-19.4 TABLE S3 OTUs with differential abundance across different storage methods. Download Table S3, XLSX file, 0.02 MB. Copyright © 2020 Liang et al. 2020 Liang et al. This content is distributed under the terms of the Creative Commons Attribution 4.0 International license.

10.1128/mSphere.00763-19.5 TABLE S4 Effects of RNALater on abundance levels of top 50 OTUs. Results are based on two-sided tests assuming equal variances. For each significant pair, the key of the smaller category appears in the category with the larger mean. Tests are adjusted for all pairwise comparisons within a row of each innermost subtable using the Bonferroni correction. Download Table S4, XLSX file, 0.01 MB. Copyright © 2020 Liang et al. 2020 Liang et al. This content is distributed under the terms of the Creative Commons Attribution 4.0 International license.

### Sampling regions and storage and retrieval methods influenced metabolite profiles.

**FIG 4:**
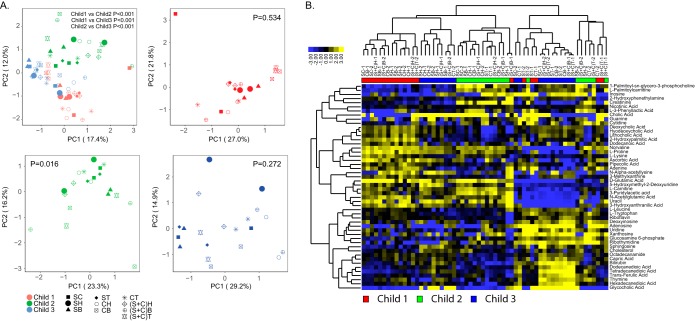
Fecal metabolites varied by subsample region. (A) PCA based on metabolite profiles across different fecal sampling sites for individual study participants combined and individually. *P* values were obtained using PERMANOVA comparing three study participants (combined study participant PCA) or surface (SH, SB, and ST) versus core (CH, CB, and CT) samples for individual participant PCA. (B) Hierarchical clustering analysis of the 50 most abundant metabolites across different fecal sampling sites.

To investigate the influence of sampling regions and storage methods on metabolite profiles, we performed ultrahigh performance liquid chromatography coupled to tandem mass spectrometry (UPLC–MS/MS) on 96 stool samples. We identified a total of 176 metabolites (Table S5). Subsequent analysis showed that the abundance levels of 22 metabolites varied significantly across different sampling locations and that there were differences between subsample locations and SC samples for only two metabolites (Table S6). PCA showed that the effect of individual variability was stronger than the effects of sampling regions on metabolome profiles (Fig. 4A; PERMANOVA *P* < 0.001 for individual effects), which was further demonstrated by clustering analysis of the 50 most abundant metabolites (Fig. 4B). A comparison of surface samples (surface head [SH], surface body [SB], and surface tail [ST]) versus core samples (core head [CH], core body [CB], and core tail [CT]) for each study participant individually (Fig. 4A) showed a significant difference for child 2 (*P* = 0.016), whereas no significant difference was observed for child 1 (*P* = 0.534) and child 3 (*P* = 0.272).

10.1128/mSphere.00763-19.6 TABLE S5 Metabolite table for different sampling regions. Download Table S5, XLSX file, 0.2 MB. Copyright © 2020 Liang et al. 2020 Liang et al. This content is distributed under the terms of the Creative Commons Attribution 4.0 International license.

10.1128/mSphere.00763-19.7 TABLE S6 Effect of subsample location on metabolite profiles. Significance of differences between groups is determined using nonparametric (Kruskal-Wallis) test. Pairwise comparisons are based on two-sided tests assuming equal variances. For each significant pair, the key of the smaller category appears in the category with the larger mean. Tests are adjusted for all pairwise comparisons within a row of each innermost subtable using the Bonferroni correction. Download Table S6, XLSX file, 0.05 MB. Copyright © 2020 Liang et al. 2020 Liang et al. This content is distributed under the terms of the Creative Commons Attribution 4.0 International license.

**FIG 5:**
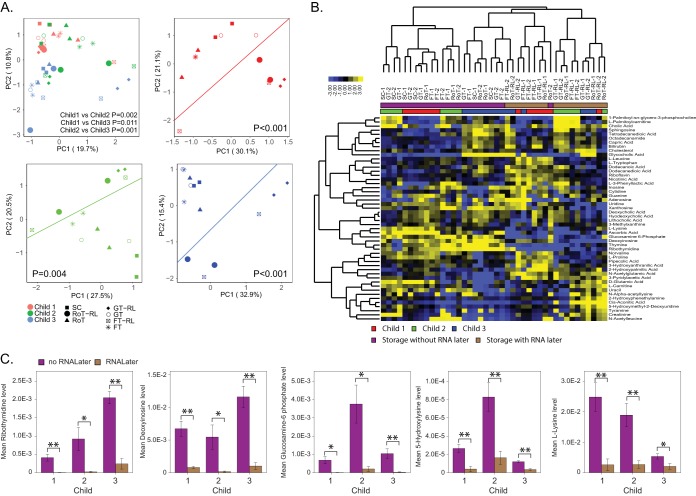
RNALater strongly impacts fecal metabolite levels. (A) PCA based on metabolite profiles for different storage and retrieval methods. *P* values were obtained using PERMANOVA comparing three study participants (combined study participant PCA) or RNALater storage (RoT-RL, GT-RL, and FT-RL) versus no RNALater storage (RoT, GT, FT, and SC) samples for individual participant PCA. (B) Hierarchical clustering analysis of the 50 most abundant metabolites across different storage and retrieval methods. (C) Relative abundance levels of metabolites for individual children’s samples separated by RNALater storage condition. Error bars represent standard errors. Asterisks indicated statistical significance (*, *P*_adj_ < 0.05; **, *P*_adj_ < 0.01).

When comparing metabolite profiles between different storage methods, we performed PCA and found significant separation between samples based on individual variability (Fig. 5A and Table S7; *P* < 0.011 based on PERMANOVA). However, PCA for each child individually showed that samples with and without RNALater were significantly different (*P* < 0.004 based on PERMANOVA; Fig. 5A), which was further demonstrated by heatmap analysis of the top 50 metabolites (Fig. 5B). The abundance levels of 26 metabolites varied significantly across different storage and retrieval methods (Table S8). To analyze the effect of RNALater on metabolite profiles in more detail, we grouped samples that were stored in RNALater and compared the metabolite levels of all 176 metabolites to the remaining samples not stored in RNALater for each child separately. We observed a significant change in 25% of metabolites. A total of 21 metabolites were significantly downregulated and 22 metabolites were significantly upregulated in samples stored in RNALater compared to samples not stored in RNALater (*P* < 0.05; Fig. 5C and Table S9). Five metabolites were consistently downregulated in samples stored in RNALater for all three children (*P* < 0.05): 5-hydroxylysine, deoxyinosine, glucosamine 6-phosphate, l-lysine, and ribothymidine (Fig. 5C and Table S9).

10.1128/mSphere.00763-19.8 TABLE S7 Metabolite table for different storage methods. Download Table S7, XLSX file, 0.1 MB. Copyright © 2020 Liang et al. 2020 Liang et al. This content is distributed under the terms of the Creative Commons Attribution 4.0 International license.

10.1128/mSphere.00763-19.9 TABLE S8 Effects of storage and retrieval methods on metabolite profiles. Significance of differences between groups is determined using nonparametric (Kruskal-Wallis) test. Pairwise comparisons are based on two-sided tests assuming equal variances. For each significant pair, the key of the smaller category appears in the category with the larger mean. Tests are adjusted for all pairwise comparisons within a row of each innermost subtable using the Bonferroni correction. Download Table S8, XLSX file, 0.04 MB. Copyright © 2020 Liang et al. 2020 Liang et al. This content is distributed under the terms of the Creative Commons Attribution 4.0 International license.

10.1128/mSphere.00763-19.10 TABLE S9 Effect of RNALater on abundance levels of metabolites. Significance of differences between groups is determined using nonparametric (Kruskal-Wallis) test. Pairwise comparisons are based on two-sided tests assuming equal variances. For each significant pair, the key of the smaller category appears in the category with the larger mean. Tests are adjusted for all pairwise comparisons within a row of each innermost subtable using the Bonferroni correction. Download Table S9, XLSX file, 0.03 MB. Copyright © 2020 Liang et al. 2020 Liang et al. This content is distributed under the terms of the Creative Commons Attribution 4.0 International license.

## DISCUSSION

Although microbial studies have increased rapidly, there is no consensus on quality control of collection and preservation of fecal specimens. However, different collection and storage methods may introduce experimental variation, especially for large-scale studies with many participants. In this study, we found that abundance levels of specific microbial families (*Bacteroidaceae*, *Bifidobacteriaceae*, *Rikenellaceae*, *Ruminococcaceae*, *Lachnospiraceae*, and *Pasteurellaceae*) changed significantly depending on fecal sampling locations. Since the surface of feces is in closer contact with the intestinal mucosa, it is possible that the variability is a reflection of the various taxa that harbor different gut microenvironments (19). It is also possible that surface samples are more susceptible to environmental factors (e.g., oxygen levels) than core samples, since some of the sampling location variable microbial families (*Bacteroidaceae* and *Bifidobacteriaceae*) are obligate anaerobic microorganisms. Gorzelak and colleagues also reported a large variation in microbes found within fecal samples and on the surfaces of fecal samples (11), although the specific microbes were inconsistent with our results. Also, this study did not further analyze the similarity of the gut microbiome structure between specific fecal locations and homogenized fecal samples. Our study showed that the alpha diversity and overall microbiome structure did not significantly differ between subsampling regions and standard control samples (homogenized and snap-frozen sample).

For field studies, the goal is to develop convenient and reliable fecal preservation methods to guarantee gut microbiome study accuracy. Previous studies have reached inconsistent conclusions about the association between microbiota composition and room temperature storage conditions. Dominianni et al. found that the microbial structure and relative abundance of major taxa did not change across different collection and storage methods, including storage at room temperature for 3 days (20), while Shaw et al. suggested that storing samples at room temperature introduced significant changes in the microbial community after 2 days (21). Our study found that the microbiome community was stable when samples were stored at room temperature for 52 h. Gradual or rapid thawing for a longer time (no more than 4 h) introduced slight changes in the measured microbiome composition. Flores et al. reported that the bacterial community composition was stable at room temperature if the samples were stored in RNALater for 7 days (22), and Sinha et al. also found that RNALater can preserve the microbiome in delayed frozen stool samples (23). However, Choo et al. found that samples stored in RNALater showed substantial divergence compared to control samples stored at −80°C (24). Our findings supported the results of Choo et al. in that RNALater significantly changed the abundance at the OTU level independent of storage and retrieval methods. Song et al. also suggested that RNALater did not protect bacteria when stool samples had been frozen and thawed (25), although details regarding the freeze-thawing were not presented in their study. RNALater can decrease DNA purity (20) and reduce the extracted DNA yield from feces (11, 26), which may lead to loss of low-abundance bacteria and a decrease in highly abundant microorganisms. Similar to other published results (20, 21, 23, 25), we also found that interindividual variation is greater than the variation introduced by sampling location and storage methods.

We found that metabolite abundance levels varied for different stool areas, but this difference did not substantially affect individual differences in metabolite profiles. We found that the abundance levels of approximately 15% of metabolites were significantly affected by storage and retrieval methods. The possible reason is that some metabolites are sensitive to temperature fluctuations or affected by changes in microbial metabolism (27). Washburn et al. (27) found that glucocorticoid metabolites increased when white-tailed deer feces were stored at room temperature for 7 days. They suggested that increased microbial metabolism may at least partially explain these results. We found that storage of stool samples in RNALater significantly affected approximately 25% of metabolites and conclude that storage in RNALater was not suitable for metabolomic studies. We hypothesize that the main component of RNALater (thiocyanate) was responsible for the extraction of nucleophilic metabolites.

### Conclusion.

We conclude that stool sample location does minimally influence the variability in microbial community abundance levels. We further conclude that homogenizing stool samples is important to reduce variability in metabolomic analysis and could also aid in reducing variability for some microbial families that vary by sampling location. Finally, we conclude that the use of RNALater as a storage medium of stool samples for microbial and metabolomic analyses is not recommended. Keeping experimental variation to a minimum is important especially for large-scale human microbiome studies across multiple collection sites and processing laboratories. On the basis of our results, we recommend that room temperature or household freezers might be an easy and temporary storage condition for reliable detection of microbial abundance levels and metabolites.

## MATERIALS AND METHODS

### Subjects and ethics.

Three healthy children aged 34 months were enrolled (one boy and two girls), who were enrolled in the same community nursery for at least 6 months prior to sample collection. Three meals and snacks were provided by the nursery each day. The primary caregiver provided standard demographic data, including age, sex, health, and physical condition. No child in this study had any antibiotic usage or illness diagnosed by clinical examination for 3 months prior to stool sample collection.

This study was conducted according to the guidelines laid down in the Declaration of Helsinki, and all procedures involving human subjects were approved by the Nanjing medical university ethics committee (FWA00001501). Written informed consent was obtained from each participant’s guardians.

### Fecal sample collection.

In the child’s home, fresh fecal samples were directly collected in a prepared sterile enamel tray. Our researchers immediately processed the whole stool for different purposes with specific tools (sterile polypropylene bag with sealing strip, sterile polyethylene tweezers, and sterile polyethylene toothed knives). We assembled a simple test bench in their bathroom, and we wiped it with 75% ethanol before the sample was processed. The fecal characteristics were normal, and each child had on average one bowel movement per day.

### Subsample preparation.

Subsamples were collected from different stool locations. Each stool sample was divided into equal parts (parts A and B) along its longitudinal axis. Overall, part A was used to identify an optimal sampling location for microbiome and metabolomic analysis. Part B was used to evaluate the effects of different storage and thawing conditions on gut microbiome community and metabolome profile and to explore the protective effect of RNALater as a collecting reagent. First, part A was equally separated into three sections according to the order of defecation (head, body, and tail), four duplicated subsamples (two 200-mg subsamples and two 100-mg subsamples) were collected from each sampling locations, including the following: (i) surface of fecal head (SH), (ii) surface of fecal body (SB), (iii) surface of fecal tail (ST), (iv) core of fecal head (CH), (v) core of fecal body (CB), (vi) core of fecal tail (CT), (vii) surface and core of fecal head [(S+C)H], (viii) surface and core of fecal body [(S+C)B], and (iv) surface and core of fecal tail [(S+C)T]. Each aliquot was rapidly put into 2.0-ml Eppendorf tubes using a sharp edge sterile spatula and spoon within 30 min and then frozen in liquid nitrogen until it was used to extract microbiota DNA or metabolites (Fig. 1).

### Sample preparation to test storage and retrieval methods.

Part B was collected into a sterile polypropylene bag with sealing strip, and then homogenized to a uniform consistency. After homogenization, four duplicated aliquots (two 200-mg aliquots and two 100-mg aliquots) were collected for each storage and retrieval regimen. Two duplicates (200 mg each) were used for studying the microbiome composition, and the other two duplicates (100 mg each) were used to analyze metabolite profiles. Details of the storage methods are described below and summarized in Fig. 1.

1. Flash-frozen in liquid nitrogen for 52 h (48 h plus 4 h) as a standard control (SC).
2. Frozen in a common household freezer (−16°C) for 48 h, then removed from freezer, and kept at room temperature for 4 hours with ice bag, as the model of gradual thawing (GT).
3. Frozen in a common household freezer (−16°C) for 48 h, then removed from freezer, and kept at room temperature for 4 h without ice, as a model of fast thawing (FT).
4. Frozen with preserving fluid (RNALater) in a household freezer (−16°C) for 48 h and then kept at room temperature on ice for 4 h as a model of gradual thawing of sample with RNALater (Life Technologies, Carlsbad, CA, USA) (GT-RL).
5. Frozen with preserving fluid (RNALater) in a household freezer (−16°C) for 48 h and then kept at room temperature without ice for 4 h as a model of fast thawing with RNALater (FT-RL).

The two sets of samples (GT-RL and FT-RL) were used to study the protective effects of RNALater on microbiome structure of thawing frozen stool samples.

6. Stored at room temperature for 52 h (48 h plus 4 h) without adding any protection (RoT).
7. Stored at room temperature for 52 h (48 h plus 4 h) in RNALater (RoT-RL).

A total of 192 stool samples were obtained for 16S and metabolomic analysis. DNA or metabolites were extracted from each subsample at the end of the storage and retrieval method.

### DNA extraction.

DNA extraction was conducted in a clean bench (Heraguard ECO clean bench; Thermo Scientific). Total DNA of feces was extracted using the Qiagen Stool Minikit according to the manufacturer’s instructions (Qiagen, Germany). The 16S rRNA gene “hypervariable region V3” was amplified using barcoded PCR primers (V3-F, CCAGACTCCTACGGGAGGCAG; V3-R, CGTATTACCGCGGCTGCTG). The PCR mixtures included approximately 5 to 10 ng DNA template, 3 μmol of each primer in 20-μl volume of TopTaq buffer containing 2 U of TopTaq DNA polymerase (Qiagen, Germany). After denaturation at 94°C for 20 s, PCR amplification was conducted for 30 cycles using the following parameters: 2 min at 94°C predenaturation, 60°C annealing for 20 s, 72°C extension for 30 s, and held at 72°C for 10 min. The concentration and purity of DNA were evaluated on 1% agarose gels. DNA was diluted to 1 ng/μl using sterile water.

### 16S rRNA gene sequencing and quality control.

PCR products were purified with GeneJET gel extraction kit (Thermo Fisher Scientific, USA). Sequencing libraries were generated using Illumina TruSeq DNA PCR-Free Library Preparation kit (Illumina, USA) following the manufacturer’s recommendations, and index codes were added. The library was sequenced on an Illumina HiSeq platform, and the paired-end reads were merged using FLASH software (V1.2.7; http://ccb.jhu.edu/software/FLASH). Low-quality and short sequences were trimmed using QIIME (28) (V1.7.0; http://qiime.org/scripts/split_libraries_fastq.html). Chimeras were removed using UCHIME software (29) (http://www.drive5.com/usearch/manual/uchime_algo.html) and Gold database (30) (http://drive5.com/uchime/uchime_download.html).

### Microbiome bioinformatic analysis.

Sequence data analysis and visualization were performed using QIIME software and R (version 3.1.1). Sequences with ≥97% identity were gathered into the same operational taxonomic units (OTUs) by Uparse *de novo* (29, 31) (Uparse v7.0.1001; http://drive5.com/uparse/). The most abundant sequences were selected as the representative sequence for each OTU. Venn diagrams were drawn using the online tool venny 2.1.0 (J. C. Oliveros, 2007 to 2015; http://bioinfogp.cnb.csic.es/tools/venny/index.html) (32). Taxonomy assignments were confirmed by mothur (33) according to the SILVA ribosomal database (34), and fast multiple sequence alignment was performed using MUSCLE (35) (version 3.8.31; http://www.drive5.com/muscle/) with default parameters. Three indices, including alpha diversity index (abundance-based coverage estimator [ACE]), Shannon, and Chao1 were applied to assess alpha diversity. A UniFrac distance matrix was calculate by QIIME to assess beta diversity (36). Dissimilarity between samples was estimated by principal-component analysis (PCA) (based on UniFrac distance matrix) and nonmetric multidimensional scaling (NMDS) analysis (based on Bray-Curtis dissimilarity values) (37). UPGMA (unweighted pair group method with arithmetic mean) clustering tree was used to analyze microbial similarity. All analysis of microbiota structure was performed on a uniform data set after rarefaction to 28,265 reads according to the sample with minimum number of reads.

### Metabolite extraction and metabolome profiling analysis.

Fecal metabolites were extracted by the method described by Meng Yu et al. (38). Frozen stool samples (100 mg) were thawed at room temperature. Ice-cold water (500 μl) was added to the sample and then homogenized by vortexing for 15 s. The homogenized samples were further disrupted using ultrasonic wave treatment for 5 min (power, 60%; four to six pulses), and the supernatant was transferred to a new 2-ml tube after centrifugation at 14,000 × *g* for 15 min. Methanol (100%) (1,200 μl) was added to the remaining pellet and treated as described above. The two supernatants were merged and filtered through an organic filtering membrane (diameter, 0.22 μm). Finally, 10 μl internal quantitative standard was mixed with the filtered solution and then volatilized to dry. The dry residue was reconstituted in 20 μl deionized water, and an aliquot of 10 μl was used for metabolite analysis.

The contents of the reconstituted samples (metabolites) were determined by ultrahigh performance liquid chromatography coupled to tandem mass spectrometry (UPLC–MS/MS) performed with a Q-exactive mass spectrometer (Waters, USA). Metabolomic analysis was performed according to our previous study (39). The column used for the chromatographic separation was a Hypersil Gold C_18_ column (100 mm by 2.1 mm; diameter, 1.9 μm; Thermo Scientific, Germany) at 40°C. The mobile phase consisted of acetonitrile containing 0.1% formic acid as mobile phase A and 0.1% aqueous formic acid (vol/vol) as mobile phase B at a flow rate of 0.4 ml/min. The gradient elution program was as follows: 0 to 3 min, 1% mobile phase A; 3 to 10 min, 1% to 99% mobile phase A; 10 to 30 min, maintained at 99% mobile phase A; and 13 to 15 min, 99% to 1% mobile phase A. The injection volume was 10 μl. The mass spectrometer was operated in the HESI (heated electrospray ionization) mode. The parameters were as follows: positive ion mode spray voltage, 3.5 kV; negative ion mode spray voltage, 2.5 kV; capillary temperature 250°C in the two modes; heater temperature, 425°C; sheath gas flow rate, auxiliary gas flow rate, and sweep gas flow rate were optimized at 50 arbitrary units (AU), 13 AU, and 0 AU, respectively; lens voltage, 60 V. The full scanning range was from 70 to 1,050 (*m/z*), and resolution was 70,000. The metabolite identification was based on the parameters of retention time to mass debris, accurate mass of standards; this process was performed using the TraceFinder 3.1 (Thermo Fisher Scientific) software platform. PCA was used for the data analysis in R (version 3.1.1).

### Statistical analysis.

For variables that exhibited a normal distribution, a two-way analysis of variance (ANOVA) test was performed before paired Student’s *t* test for multiple comparisons. For variables with a skewed distribution, the nonparametric Friedman test was used. For comparisons that reached statistical significance, a q test was employed to correct for multiple comparisons. Permutational multivariate analysis of variance (PERMANOVA) was used to assess microbiota or metabolite variability explained by the corresponding variables. The nonparametric Kruskal-Wallis test was used to determine effects of storage and retrieval methods and RNALater on metabolite profiles. All analyses were carried out using R software and SPSS, and statistical significance level was *P* < 0.05.

### Data availability.

All sequences are available under the NCBI Sequence Read Archive BioProject identifier (ID) PRJNA579560.
