# Supplementary figures and images for: Systematic Analysis of Impact of Sampling Regions and Storage Methods on Fecal Gut Microbiome and Metabolome Profiles
Source: mSphere. 2020 Jan 8;5(1):e00763-19. doi: 10.1128/mSphere.00763-19 (PMC6952195; doi:10.1128/mSphere.00763-19)

Supplementary Figure 1

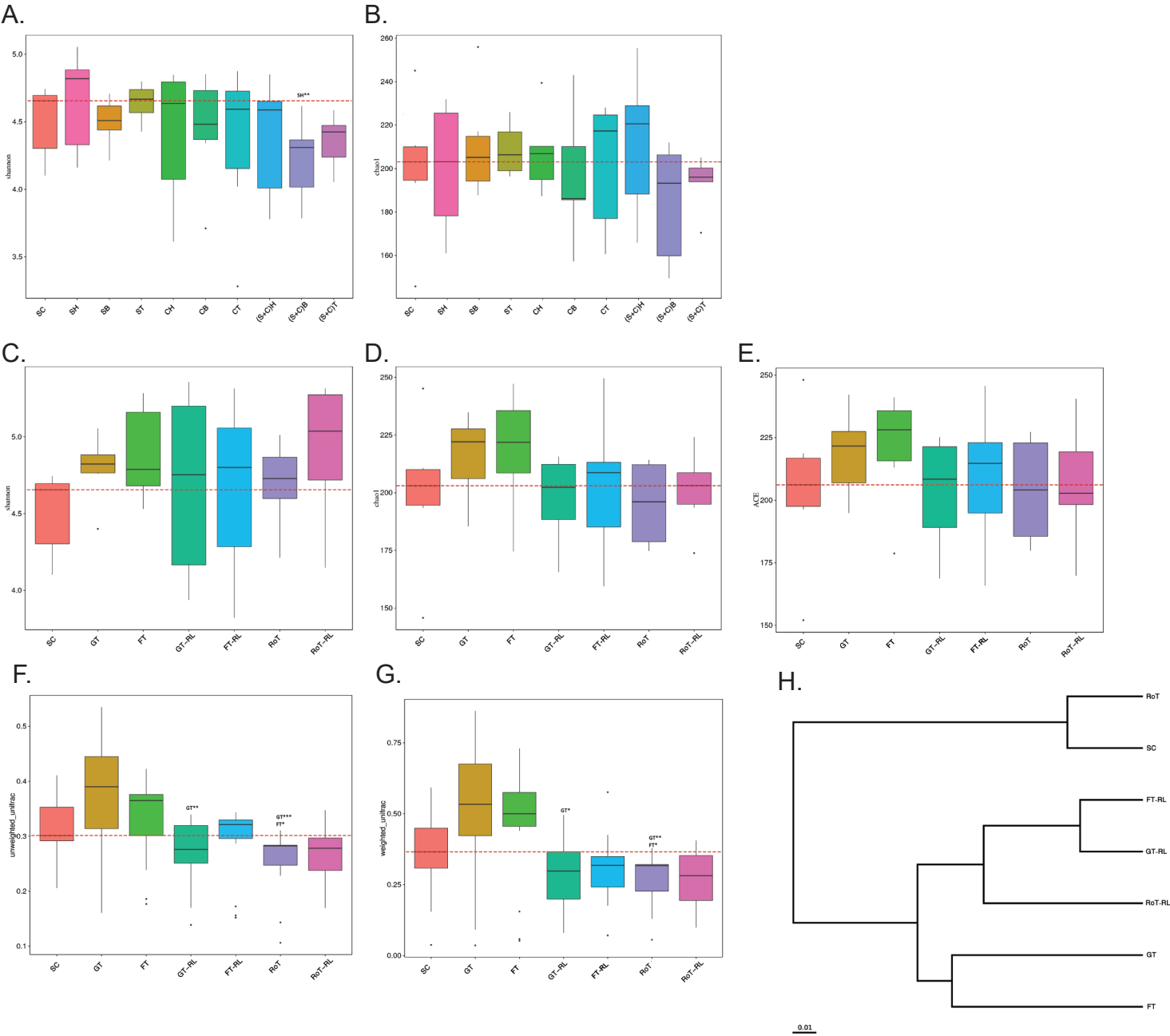

Supplement: FIG S1 [file mSphere.00763-19-sf001.pdf]
